# The pivotal role of tertiary lymphoid structures in the tumor immune microenvironment

**DOI:** 10.3389/fonc.2025.1616904

**Published:** 2025-05-22

**Authors:** Chengsen Liu, Jiandong Cao

**Affiliations:** ^1^ Department of Radiotherapy, The People’s Hospital of Liaoning Province, Shenyang, Liaoning, China; ^2^ Department of Thoracic Surgery, Shenyang Chest Hospital and Tenth People’s Hospital, Shenyang, Liaoning, China

**Keywords:** tertiary lymphoid structure, TLS, immunotherapy, tumor immune microenvironment, TME

## Abstract

Tertiary lymphoid structures (TLS) are ectopic lymphoid structures that form in non-lymphoid tissues in response to chronic inflammatory stimulation. Structurally and functionally resembling lymph nodes, TLS are primarily composed of B cells, T cells, dendritic cells, and other immune cell populations. Critically, TLS serve as direct sites for initiating anti-tumor immune responses. Within tumors, TLS facilitate the accumulation of immune cells—particularly effector subsets such as cytotoxic T cells and antibody-producing B cells—in the tumor microenvironment, thereby establishing a localized hub for both cellular and humoral immunity. This localized immune activation correlates with improved patient prognosis and enhanced responses to immunotherapy. In this review, we summarize the organization, formation drivers, detection markers, and the interplay between TLS and tumor-associated genes. Furthermore, we discuss the potential of TLS as biomarkers for immunotherapy efficacy and their translational clinical applications.

## 1 Components of TLS

**Figure 1:**
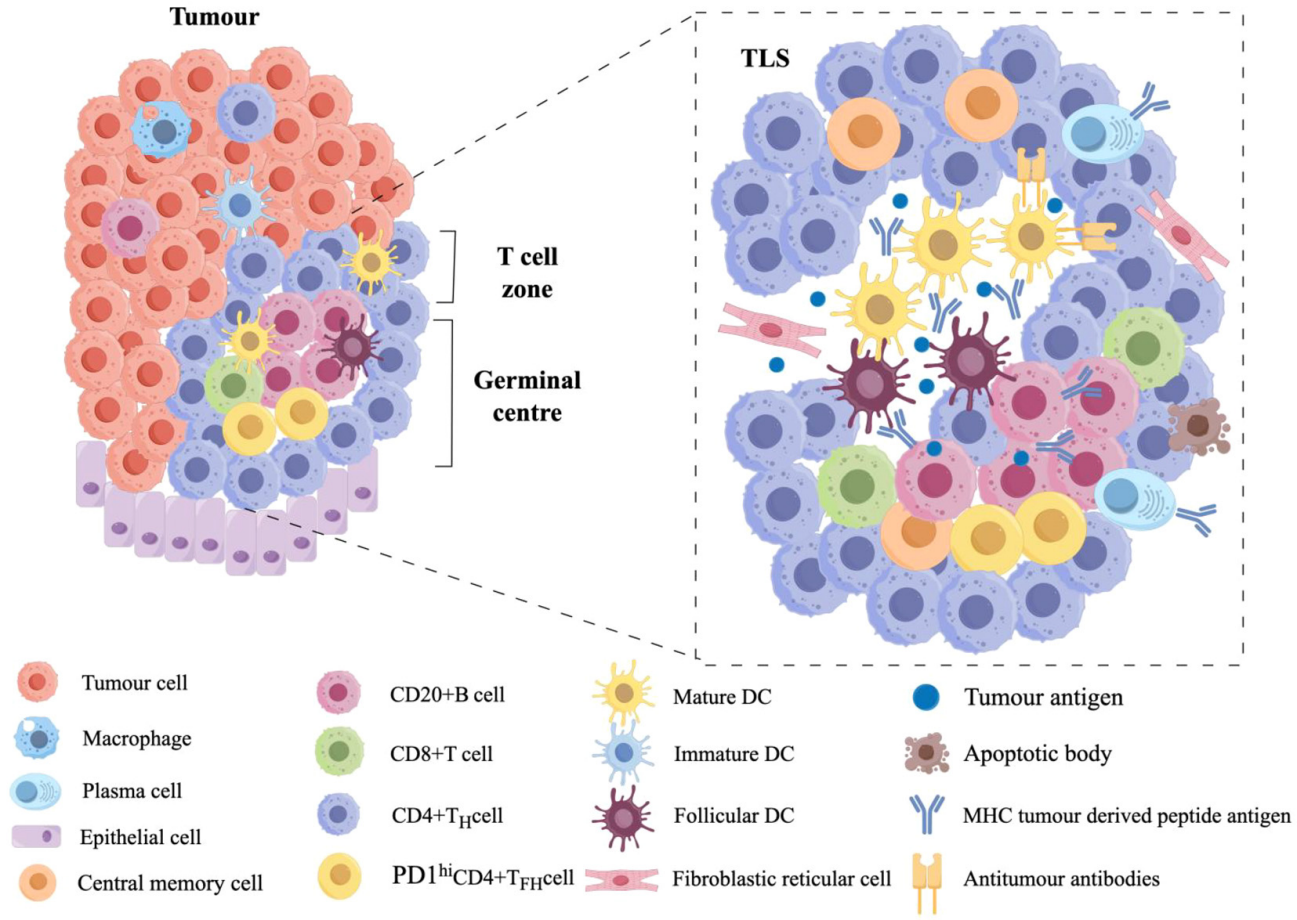
The structural composition of TLS (Tertiary Lymphoid Structures) in the tumor microenvironment. The TLS primarily contains B cells, follicular dendritic cells (FDCs), T cells, fibroblastic reticular cells (FRCs), stromal cells, dendritic cells, neutrophils, macrophages, and endothelial cells. Among these, FDCs are typically located in the B-cell zone, expressing various receptors to facilitate antigen presentation to B cells, thereby promoting the production of highly active antibodies. Endothelial cells are situated at the periphery of the TLS. Additionally, within the T-cell zone, there are some FRCs that interact with the local microenvironment to regulate T-cell function.

Tertiary lymphoid structures (TLS) comprise diverse immune and stromal components, including B cells, T cells, follicular dendritic cells (FDCs), fibroblastic reticular cells (FRCs), stromal cells, dendritic cells (DCs), macrophages, and endothelial cells (1, 2). Within this organized architecture, FDCs predominantly localize to B cell zones, where they express an array of surface receptors to efficiently present antigens and orchestrate B cell differentiation into antibody-producing cells (3). Meanwhile, endothelial cells occupy TLS peripheries and T cell-rich regions, collaborating with fibroblasts to modulate T cell activity. Together, these spatially and functionally distinct cellular interactions not only establish the structural foundation of TLS but also sustain immune responses within the tumor microenvironment (TME) (4, 5)(**Figure 1**).

### 1.1 B cells in TLS

**Figure 2:**
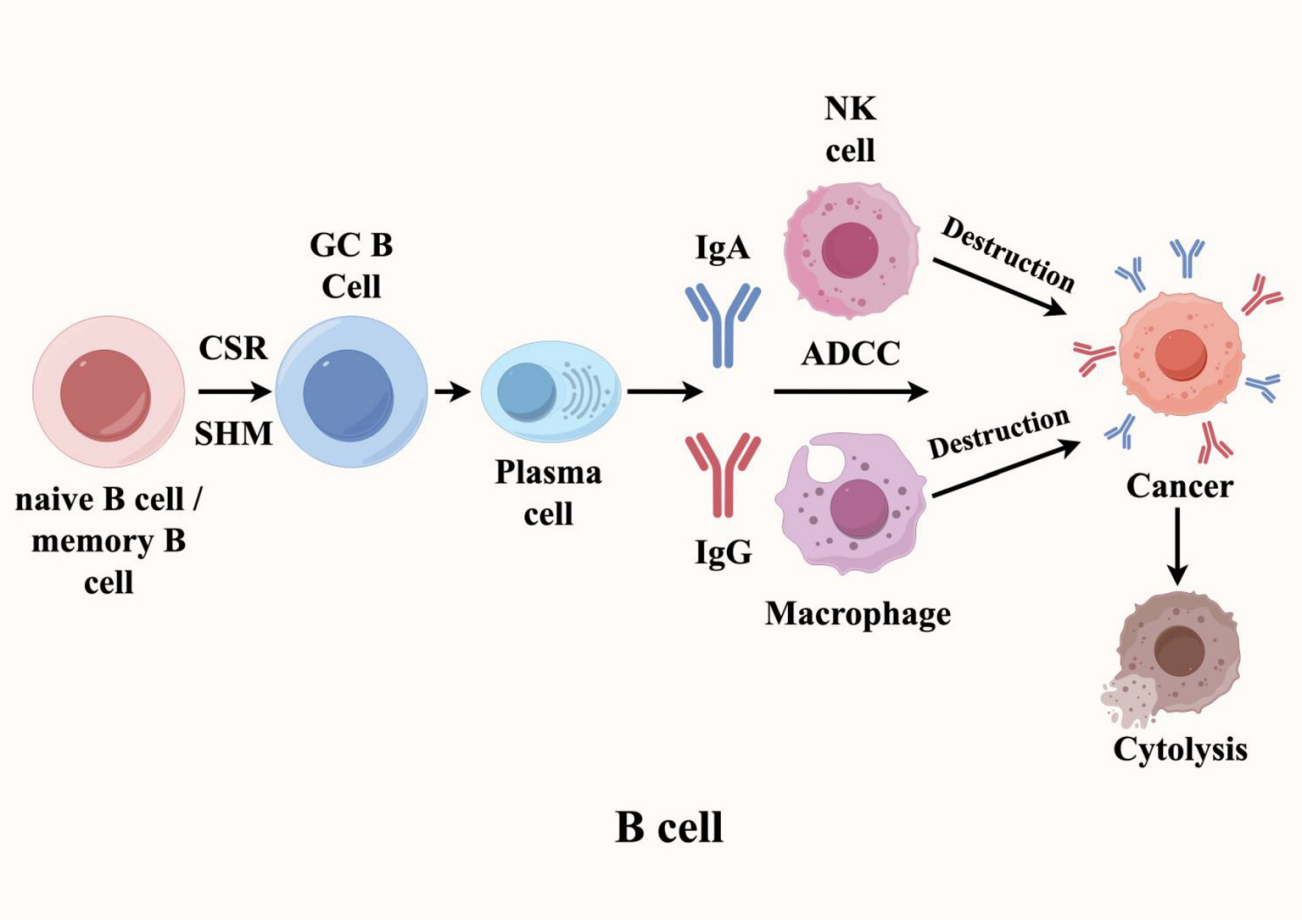
B cells play a dual role in TLS (Tertiary Lymphoid Structures), exhibiting both anti-tumor and tumor-promoting effects. In the tumor microenvironment, when B cells within TLS encounter tumor-associated antigens, they differentiate into memory B cells and plasma cells through the synergistic actions of somatic hypermutation (SHM) and class-switch recombination (CSR). The plasma cells then secrete high-affinity IgG and IgA antibodies targeting specific antigens on the surface of tumor cells, thereby inducing antibody-dependent cellular cytotoxicity (ADCC).

In antitumor humoral immunity, the orchestrated activation, proliferation, and differentiation of B cells constitute a critical effector mechanism. Within mature TLS, class switch recombination (CSR) and somatic hypermutation (SHM) are the main events of B cell activation. The resulting high-affinity IgG/IgA antibodies specifically bind to tumor cell antigens, triggering antibody-dependent cellular cytotoxicity (ADCC) and thereby enhancing anti-tumor immunity (6–13). For instance, in renal cell carcinoma, Meylan et al. demonstrated that tumors with high TLS signature gene expression exhibited markedly elevated clonal indices for immunoglobulin heavy (IgH) and light chains (IgL), with increased IgH clonality showing particular prominence compared to TLS-low tumors (14). These findings reveal antigen-driven B cell selection within TLS, accompanied by robust SHM and CSR activity that culminates in plasma cell differentiation. Taken together, these data position TLS as pivotal hubs for B cell maturation into either memory B cells or antibody-secreting plasma cells, thereby establishing tumor-targeted humoral immunity through IgG/IgA production (**Figure 2**).

Clinical evidence further underscores the prognostic significance of TLS-associated humoral immunity. In head and neck squamous cell carcinoma (HNSCC), the presence of IgA- and IgG1-secreting plasma cells within TLS correlates strongly with improved patient outcomes (15). Mechanistically, studies in ovarian cancer mouse models reveal that T follicular helper (Tfh) cells orchestrate antibody class switching in TLS-resident B cells, thereby amplifying their antitumor potency. Moreover, across diverse malignancies—including cutaneous melanoma, omental ovarian cancer metastases, and gastroesophageal adenocarcinoma—B cells within tumor-associated TLS exhibit substantially more pronounced immunoglobulin gene rearrangement and clonal expansion than their peripheral counterparts (16–20). Together, these consistent observations highlight that antigen-driven B cell activation and proliferation within TLS represent a hallmark feature of productive antitumor immunity.

However, the TME harbors immature TLS that may foster B cell differentiation into regulatory B cells (Bregs). These immunosuppressive Bregs secrete cytokines like TGF-β, actively remodeling the immune landscape to promote tumor immune evasion (21–23). Notably, the functional impact of TLS exhibits striking cancer-type specificity—while in prostate cancer, a unique plasma cell subset suppresses CD8^+^ T cell activity, other tumor types may experience polyclonal B cell activation that propels macrophage polarization toward an immunosuppressive phenotype (24). Through these multifaceted mechanisms, TLS-resident B cells dynamically modulate the TME, ultimately dictating immunotherapy responses (25–27). This context-dependent functional plasticity underscores the dual roles of B cells in tumor immunity, ranging from effector responses to immunosuppression.

### 1.2 T cells in TLS

**Figure 3:**
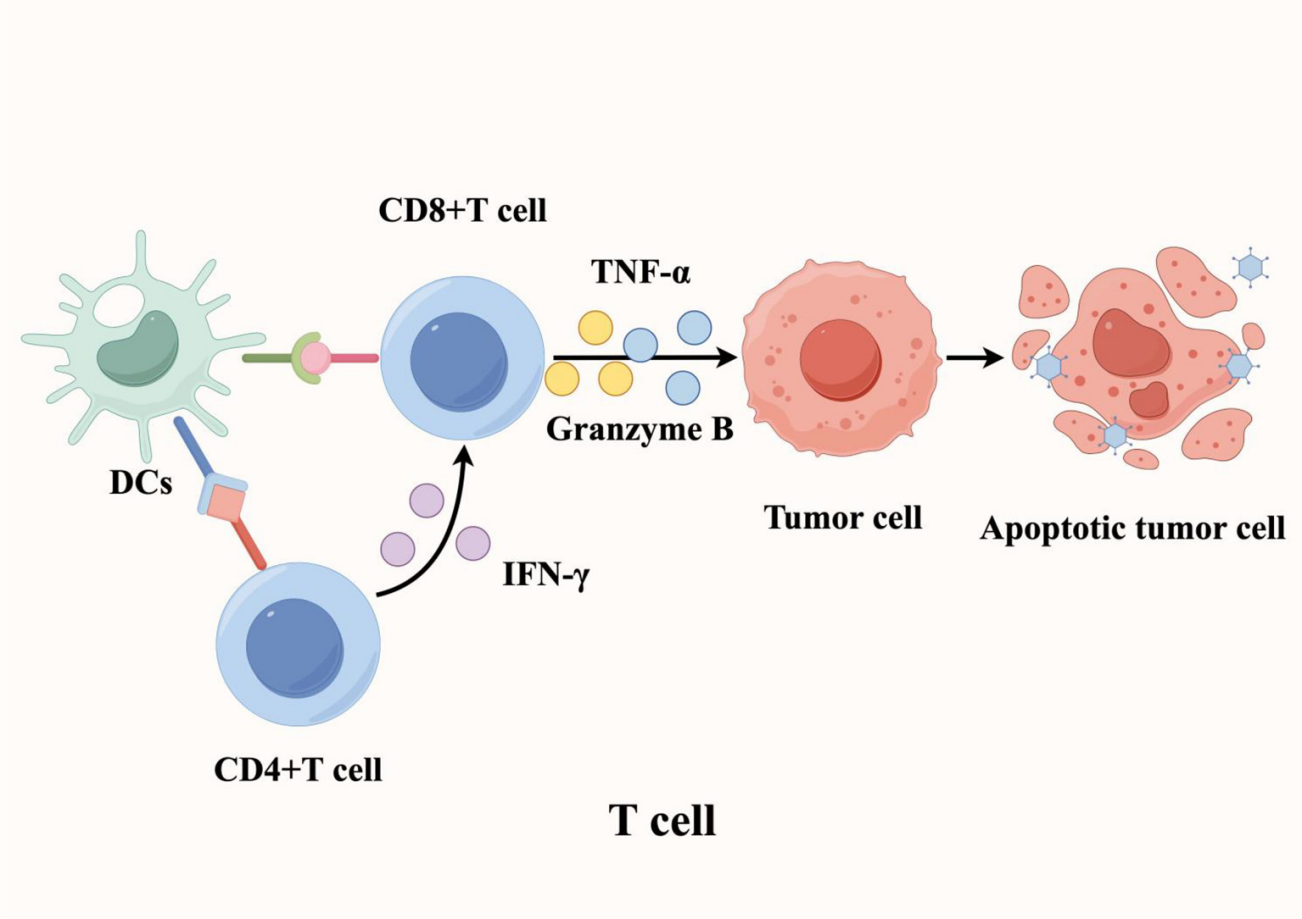
T cells play a crucial role in mediating anti-tumor immunity within tertiary lymphoid structures. Antigen-presenting cells (APCs), such as dendritic cells (DCs), process and present tumor-specific antigens to T cells, triggering their activation, proliferation, and differentiation. This process ultimately enhances tumor-targeted cellular immune responses and promotes tumor cell death.

The TME hosts immune cells with multiple functional capacities, where T lymphocytes serve as the central mediators of anti-tumor immunity. As such, their activity within the local microenvironment fundamentally determines immunotherapy outcomes (28, 29). Within TLS, a specialized immune niche, antigen-presenting cells (APCs) - particularly DCs - prime T cell responses by presenting tumor-specific antigens, driving their activation, clonal expansion, and effector differentiation. This cascade culminates in potent cellular immunity against tumors. Specifically, CD8^+^ cytotoxic T lymphocytes (CTLs) directly eliminate tumor cells through dual mechanisms: (1) release of cytotoxic granules (e.g., granzyme B) and (2) secretion of pro-apoptotic cytokines (e.g., TNF-α) (30). Meanwhile, CD4^+^ T helper cells amplify immune responses by producing IFN-γ, which enhances CTL function (31). Importantly, a subset of activated T cells differentiates into memory T cells, establishing durable immunological memory that enables rapid tumor antigen recall responses (**Figure 3**).

Research indicates that the vast majority of T cells within TLS in lung cancer tissues are effector memory T cells, with only a small number being central memory T cells and other T cell subsets (32–34). Notably, researchers also observed that CD8^+^ T cells within TLS in the TME exhibit significant cytotoxic characteristics. This observation aligns with findings across multiple cancer types—such as lung cancer, colorectal cancer (CRC), and pancreatic cancer—where the density distribution of TLS in tumor tissues is positively correlated with T cell infiltration (35–37). Additionally, in HNSCC, the Tfh cell signature gene set is associated with a favorable prognosis, further underscoring the functional importance of TLS (38). Beyond cytotoxic CD8^+^ T cells, TLS is enriched with CD4^+^ T cells skewed towards the Th1 phenotype and regulatory T cells (Tregs) with immune regulatory functions (39). These findings reveal a complex and dynamic immune cell network within TLS, where the infiltration of cytotoxic CD8^+^ T cells suggests their key role in combating tumors. In parallel, the presence of Th1-skewed CD4^+^ T cells further enhances this immune response by promoting the effector mechanisms of the anti-tumor immune reaction. Crucially, as an organized structure, TLS allows T cells to directly interact with tumor edge cells to exert antitumor effects. In contrast, in patients with diffuse immune cell distribution, macrophages infiltrating the tumor tissue increase the enrichment of Ki-67^+^ Tregs, which in turn inhibit the immune function of T cells. This contrast confirms that TLS plays a more important antitumor role compared to diffusely distributed immune cells. In summary, within the TME, TLS functions as a structure similar to secondary lymphoid organs (SLOs), capable of regulating the proliferation and differentiation of T cells, thereby exerting potent antitumor immune responses.

### 1.3 FDCs in TLS

**Figure 4:**
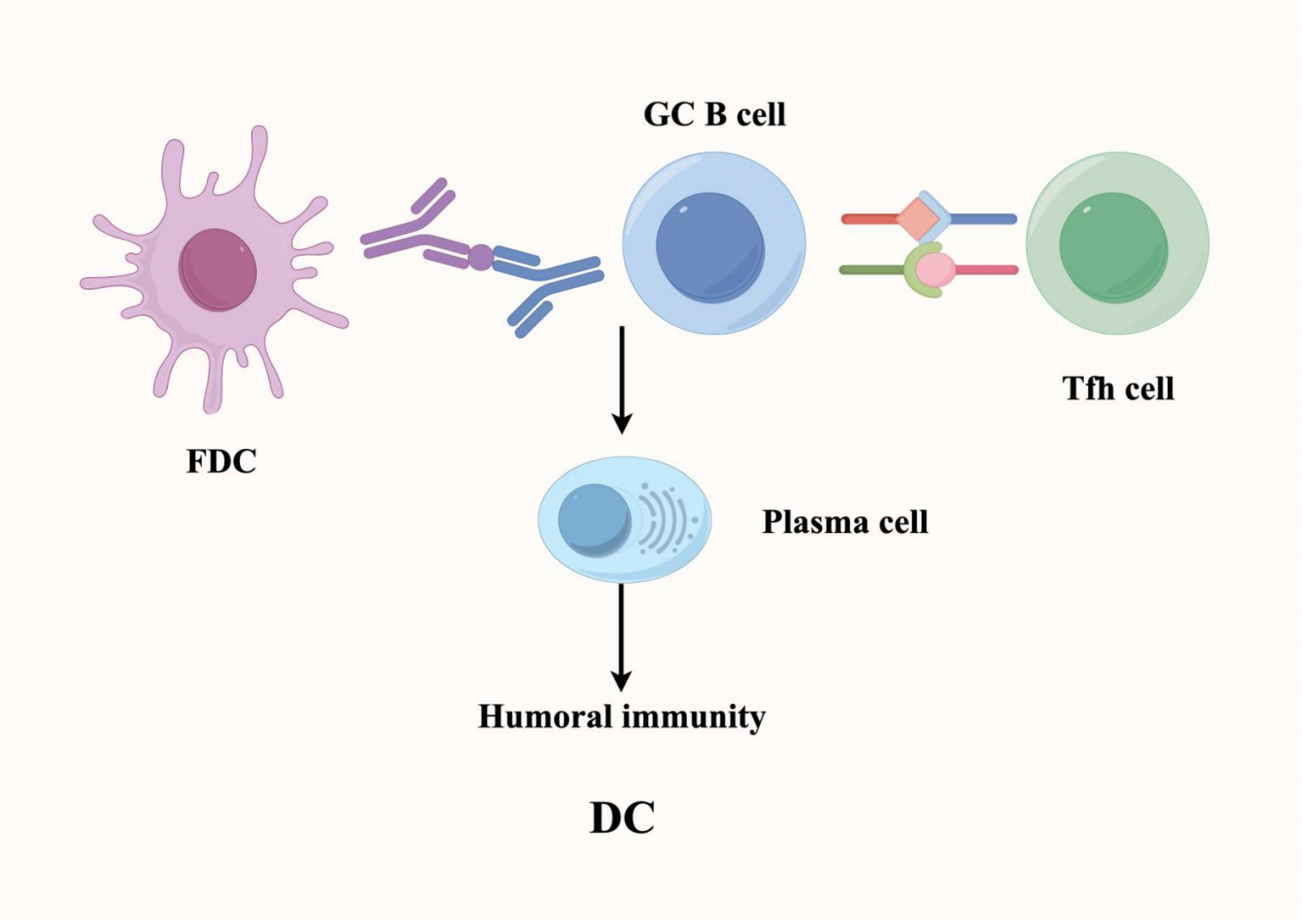
Within tertiary lymphoid structures, follicular dendritic cells (FDCs) sustain B cell-mediated humoral immune responses and maintain immunological memory. FDCs provide critical signaling cues and cellular support to promote B cell differentiation into antibody-secreting plasma cells. These plasma cells produce high-affinity antibodies targeting tumor-associated antigens, thereby enhancing tumor-specific humoral immune responses.

In the TME, tumor-associated mature TLSs are similar to SLOs, with the FDCs within them serving as a key subpopulation of cells that regulate humoral immunity. Specifically, FDCs in the TME are a special type of DC, mainly distributed in the B cell area and germinal center (GC) regions of TLSs, where they play a crucial role in B-cell mediated/humoral responses (40) (**Figure 4**). Within TLSs, FDCs interact with B cells through specific surface molecules such as CD21, CD23, and CD35, thereby promoting B cell activation, proliferation, and differentiation. Beyond direct cell–cell contact, FDCs can also present antigens to B cells, assisting them in recognizing tumor-specific antigens and facilitating B cell class switching and high-frequency mutation (somatic hypermutation) (41). Additionally, FDCs in TLSs can store and present antigens, a process that helps induce the formation of immunological memory. This memory function ensures that upon re-exposure to the same tumor antigen, memory B cells are rapidly reactivated to mount an effective and specific antibody response.

Moreover, in TLSs, the signals and cytokines provided by FDCs promote the differentiation of B cells into plasma cells, which then produce high-affinity antibodies against tumor antigens, ultimately enhancing the humoral immune response against tumors. Supporting their clinical relevance, in small cell renal cancer, increased infiltration of DCs in TLSs is closely related to lower recurrence and mortality risks (42). Furthermore, the density of DC infiltration in TLSs is negatively correlated with the infiltration of exhausted T cells in the tumor, suggesting a protective role of FDC-mediated immunity. In addition to their role in humoral regulation, FDCs recruit immune cells such as T cells, B cells, and macrophages into TLSs by secreting cytokines and chemokines, thereby helping maintain the structure and function of TLSs. This coordinated recruitment enhances local immune surveillance and immune response, further solidifying TLSs as critical regulators of antitumor immunity (43).

### 1.4 High endothelial venule and TLS

In the TME, high endothelial venules (HEVs) are key structures for the formation, maturation, and functional maintenance of TLS, and also serve as structural markers for TLS identification (44–46). Functionally, HEVs provide a rapid conduit for immune cells, enabling efficient recruitment of T cells, B cells, and other immune cells into the TLS compartment. This organized migration allows these immune cells to engage in direct interactions with tumor cells or other immune subsets, thereby enhancing immune surveillance and potentiating antitumor immune responses. Through this mechanism, HEVs facilitate the dynamic accumulation of effector lymphocytes at the tumor site, significantly enhancing tumor recognition and elimination by the immune system.

Clinically, the presence of HEVs in TLS shows a strong positive correlation with the degree of immune cell infiltration in the tumor, particularly for effector populations such as T cells, B cells, and macrophages (47). This association explains why HEV development is indicative of a favorable prognosis for cancer patients (48). Importantly, research has demonstrated that across multiple malignancies—including melanoma, head and neck squamous cell carcinoma, and breast cancer—HEV formation in TLS correlates with the activation and proliferation of immune cells within TLS microenvironments.

In summary, through their dual roles in immune cell recruitment and activation, HEVs not only contribute to the formation and structural integrity of TLS but also establish an immunologically active antitumor microenvironment, thereby amplifying the immune system’s surveillance and cytotoxic capacity against tumor cells (49).

## 2 The driving mechanism of TLS

SLOs are distributed throughout the body, including major sites such as lymph nodes, spleen, tonsils, Peyer’s patches, and mucosa-associated lymphoid tissue (MALT). These organized structures enable the immune system to sample antigens from various tissues and facilitate coordinated interactions between different immune cells, thereby promoting the induction of adaptive immune responses. Interestingly, under chronic inflammatory conditions, lymphoid tissue can undergo extranodal seeding and form TLSs at the site of inflammation (50). To better understand the development of these ectopic lymphoid aggregates, they can be structurally and functionally compared with the formation of SLOs during embryogenesis.

The seeding and organization of SLOs, particularly lymph nodes and Peyer’s patches, are initiated by a series of highly orchestrated events involving interactions between hematopoietic cells and non-lymphoid stromal cells. These interactions are mediated by cytokines, chemokines, adhesion molecules, and survival factors, which collectively regulate the spatial and temporal progression of SLOs development. The formation of SLOs begins early in embryonic development when hematopoietic lymphoid tissue inducer (LTi) cells, characterized by the expression of transcription factors RORγt and Id2, colonize the lymph node primordium. These LTi cells, which differentiate from fetal liver precursors, express lymphotoxin α1β2 (LTα1β2), which binds to the lymphotoxin β receptor (LTβR) on mesenchymal lymphoid tissue organizer (LTo) cells. This interaction drives the initial steps of SLOs formation, inducing the expression of adhesion molecules such as vascular cell adhesion molecule 1 (VCAM1), intercellular adhesion molecule 1 (ICAM1), mucosal addressin cell adhesion molecule 1 (MAdCAM1), and peripheral node addressin (PNAd), as well as the production of lymphoid chemokines including CCL19, CCL21, and CXCL13. These molecules collectively regulate the recruitment of immune cells to the lymphoid niche and the development of HEVs. The subsequent compartmentalization of newly formed lymphoid follicles is achieved through the segregated expression of homeostatic chemokines, such as CCL19+ and/or CCL21+ FRCs and CXCL13+ FDCs, which guide the distribution of lymphocytes expressing corresponding CCR7 and CXCR5 receptors, thereby enabling the formation of distinct T cell and B cell zones. Notably, the positive feedback loop induced by lymphoid chemokine secretion is essential for maintaining the lymphoid niche, as signaling through CXCR5 expressed on B cells and LTi cells can induce LTα1β2 expression (51, 52).

TLS exhibit significant anatomical similarities to SLOs, although most TLS in tissues lack a surrounding capsule. This structural distinction may allow their cellular components to directly interact with the surrounding tissue but also potentially exposes resident immune cells in TLS to large molecules in the inflammatory microenvironment. While the formation of TLS and SLOs was initially thought to be induced by the same molecular factors, such as LTα1β2-LTβR signaling and local expression of adhesion molecules and lymphoid chemokines, recent studies suggest that the cell types involved are not identical, and the precise stimuli driving TLS formation remain unclear. Additionally, several TLS molecular inducers independent of lymphotoxin signaling have been reported. Importantly, much of our understanding of the cellular and molecular processes driving TLS formation has been derived from autoimmune disease and chronic infection models, highlighting the need for caution when extrapolating these findings to other physiological or pathological contexts.

Regarding the upstream initiation mechanisms of TLS, it is currently unclear whether classical LTi cells are required to initiate local mesenchymal accumulation, or whether locally aggregated immune cells can substitute for LTi cells. Several immune cell subsets that may fulfill this role mainly include Th17 cells and Innate Lymphoid Cell 3 (ILC3), which have been observed in contexts such as allograft rejection, autoimmunity, chronic inflammation, or cancer in both mice and humans. Notably, unlike the formation of SLOs, the induction of TLS may not always depend on lymphotoxins. For example, Interleukin 17 (IL-17) produced by T cells can induce the expression of CXCL13 and CCL19 in mouse stromal cells, thereby promoting the formation of inducible Bronchus-Associated Lymphoid Tissue (iBALT), a type of TLS that forms in lung tissue. Consistent with this lymphotoxin-independent pathway, lymphoid aggregates do form in LTα-/- mice, although these structures exhibit impaired organization, lacking clear separation of T and B cell zones and functional HEVs, and thus may not be considered typical TLS. To further elucidate the role of the local tissue environment in determining the composition of TLS, studies have demonstrated that transgenic expression of different cytokines and chemokines in mouse models can induce TLS with distinct characteristics. For example, tissue-specific expression of CXCL13 induces B cell aggregates that are lacking FDC networks, while expression of TNF and CXCL12 induces small lymphocyte infiltrates, mainly composed of B cells and scattered T cells. Moreover, although CCL19 and CCL21, the ligands of CCR7, induce aggregates of similar composition, the structures induced by CCL21 expression are larger and more organized, suggesting a dose- or context-dependent effect on lymphoid neogenesis (53).

Huge differences in TLS components have been detected across various human cancers. For example, DC-LAMP+ DCs are frequently observed in the TLS of NSCLC but are rare in other cancer types. Similarly, Tfh cells have primarily been documented in the TLSs of breast cancer (54). However, these findings must be interpreted cautiously, as most available data derive from studies employing inconsistent TLS markers. Consequently, there is still a lack of standardized parameters for rigorous assessment and large-scale analysis of TLS heterogeneity among cancers. Emerging evidence suggests that TLS heterogeneity correlates with the degree of TLS maturation. For instance, based on structural similarity with SLOs, three maturation stages have been identified in NSCLC, hepatocellular carcinoma (HCC), and CRC (55). The least organized stage comprises dense lymphocyte aggregates without FDCs or distinct T/B cell zones. Primary follicle-like TLSs contain FDCs but lack GC reactions, whereas fully mature secondary follicle-like TLSs exhibit active GCs, indicating functional maturity (56). Although numerous factors influencing TLS formation have been characterized, the precise molecular determinants of the TME that promote or suppress TLS formation remain incompletely understood. To clarify this, we propose a framework categorizing TMEs where TLSs are absent: (1) a “restrictive” environment, where TLS formation is actively inhibited, and (2) an “insufficient” environment, lacking essential drivers (e.g., antigens). In summary, TLS formation and development involve a multi-step, dynamic process. Pro-inflammatory signals from immune cells initiate TLS induction, while activated fibroblasts serve as organizers, establishing lymphocyte structures and secreting chemokines to recruit cells. Through these interactions, TLSs differentiate, mature, and ultimately exert immune effects.

## 3 TLS markers and detection

Currently, TLS have been identified in various tumor types, including lung cancer, pancreatic cancer, CRC, and melanoma. However, due to the lack of standardized marker strategies, significant differences exist in TLS detection across cancer types, which complicates direct cross-cancer comparisons. To assess TLS presence and composition, commonly used methods include: hematoxylin and eosin staining (H&E), immunohistochemistry (IHC), multiplex immunohistochemistry (mIHC)/multiplex immunofluorescence (mIF), and sequencing-based assays.

## 4 Tumor-associated genes and TLS

### 4.1 EGFR mutations and TLS

In 2016, Mansuet-Lupo et al. first reported that EGFR mutations were more frequent in lung adenocarcinoma (LUAD) patients with high-density TLS (57). Subsequent studies confirmed this finding, demonstrating that EGFR mutations were enriched in TLS containing mature dendritic DCs and served as a key determinant of the tumor immune microenvironment (TIME) (58). These mutations may influence immune responses by modulating cytokine and chemokine secretion, thereby promoting T cell-mediated protective immunity.

However, the relationship between EGFR mutations and TLS remains controversial (59, 60). While some studies support this association, others found no significant difference in EGFR mutation rates between LUAD patients with or without TLS. Notably, Feng et al. observed that regardless of EGFR mutation status, patients with high TLS density had significantly better overall survival (OS) and progression-free interval (PFI) than those with low TLS density, suggesting TLS density may be an independent prognostic factor.

Furthermore, Gu et al. investigated ethnic differences in the relationship between tumor mutational burden (TMB) and genetic mutations in LUAD (61). Their findings revealed that EGFR-mutant LUAD patients of Chinese descent had better prognosis compared to Caucasian patients, highlighting the importance of ethnic considerations in LUAD prognosis. Although TMB has emerged as a potential biomarker for LUAD, its predictive value requires further validation due to inconsistent findings across studies.

### 4.2 HER-2 and TLS

Liu et al. conducted a retrospective analysis of 248 invasive breast cancer cases, revealing that HER2-positive tumors were significantly more likely to exhibit TLS expression (62). Their findings demonstrated that the HER2-positive cohort displayed: (1) increased infiltration of mature FDCs, and (2) elevated expression of lymphoid chemokines (CCL19, CCL21, and CXCL13) known to be critical for TLS formation and maintenance. A subsequent meta-analysis reinforced these observations, confirming a positive correlation between TLS presence and HER2 mutation status while additionally associating TLS with improved clinical outcomes, including both disease-free survival (DFS) and overall survival (OS) (63).

Further investigations in HER2-positive breast cancer patients undergoing surgical treatment revealed significant correlations between TLS characteristics (both density and maturity) and HER2-related parameters (immune scores and gene copy numbers) (64). Intriguingly, comparative analysis showed that the high-TLS group exhibited marked upregulation of key immune checkpoint genes relative to the low-TLS group. Based on these findings, the researchers proposed two potential mechanistic explanations: First, HER2 protein overexpression or related mutations may serve as immunogenic factors that actively recruit lymphocytes and facilitate TLS formation. Second, HER2+ ductal carcinoma *in situ* (DCIS) might promote macrophage infiltration, thereby enhancing antigen presentation and immune activation processes essential for TLS development. Notably, this association appears to be tumor type-specific, as no similar relationship between TLS and HER2 mutations has been observed in LUAD studies. This distinction underscores the importance of considering cancer-specific microenvironments when evaluating TLS formation mechanisms.

### 4.3 TP53 and TLS

In lung adenocarcinoma (LUAD), tumors with TP53 mutations exhibit higher CD8+ T cell density, mature DC infiltration, and enhanced immunogenicity. Despite these immunogenic features, no direct correlation has been found between TP53 mutations and TLS expression in LUAD. This contrasts with other cancers: for example, CRC, gastric cancer, and oral squamous cell carcinoma (OSCC) show a negative correlation between TLS presence and TP53 mutations, whereas breast cancer and low-grade gliomas with higher TLS scores tend to have a higher frequency of TP53 mutations. These divergent observations suggest a context-dependent role of TP53 in TLS regulation (65, 66). A possible explanation is that TP53-mutant tumors upregulate chemokines (CXCL9, CXCL10, CXCL11), which promote T cell effector functions and memory B cell phenotypes, thereby driving TLS formation in certain tumor types but not others.

### 4.4 BRAF and TLS

In lung cancer, BRAF mutations do not significantly influence the composition of the TIME. In contrast, in non-metastatic colorectal cancer (nmCRC), BRAF mutation status is associated with TLS formation, which follows a sequential maturation process that ultimately leads to GC development. Notably, BRAF mutations positively correlate with TLS density and maturity, particularly in BRAF V600E-mutant tumors, which exhibit higher TLS numbers (67). Meanwhile, in melanoma, approximately 66% of cases harbor BRAF mutations, yet the relationship between BRAF and TLS remains poorly characterized (68). Here, the BRAF V600E mutation regulates IL-1α/β transcription in melanocytes, leading to upregulation of immunosuppressive genes (e.g., PD-1 ligands, COX2) in cancer-associated fibroblasts. Preclinically, BRAF inhibition suppresses IL-1α production in melanoma cell lines, implying a potential indirect role in TLS modulation. However, a study of 177 melanoma cases found no correlation between B-cell transcriptional signatures (a TLS marker) and BRAF mutation status, further underscoring the context-dependent nature of BRAF-TLS interactions (69, 70).

### 4.5 BRCA and TLS

The genes encoding BRCA1/2 are involved in the detection and repair of DNA alterations and are frequently mutated in tumors such as breast or ovarian cancer. Consistent with this role, LIN et al. found that in most tumors, including breast, prostate, and endometrial cancers, many mutations (such as BRCA mutations) are positively correlated with TLS scores (71). Specifically, in ovarian cancer, HER-2/neu+ tumors, and triple-negative breast cancer (TNBC), BRCA-mutated tumors exhibit strong CD8+ T-cell infiltration (72). Supporting this observation, Solinas et al. detected an average of 4.5 TLS per square centimeter of tumor tissue in 75% of TNBC cases (73). While in BRCA-mutated tumors, tumor-infiltrating lymphocytes (TILs) were observed, with some studies suggesting that TILs are the strongest independent predictor of TLS, the study showed no difference in the density, location, or composition of TLS between BRCA-mutated and non-mutated TNBC cohorts. This discrepancy may be because mutations arising from DNA repair defects are largely independent of immune status, while TLS quantity is indirectly influenced by this process and occurs stochastically (74).

Furthermore, other studies have found that in BRCA1/2 protein-positive breast cancer tissues, the proportion of Treg infiltration in the local microenvironment decreases. Since Tregs suppress T-cell and DC-cell responses, reducing HEV formation and CCL21 expression, their reduction could diminish the inhibitory effect on TLS formation (75, 76). However, contrasting these findings, in high-grade serous ovarian cancer, no correlation was found between TLS and mutation load, BRCA1/2 status, or differentiation antigens.

### 4.6 DNA methylation and TLS

By conducting DNA methylation analysis on ccRCC samples with different TLS statuses, significant differences in methylation profiles were observed (77). Notably, in CRC, the TLS-positive group exhibited the CpG island methylator phenotype (CIMP)-high subtype. Further supporting this observation, GO enrichment analysis revealed that TLS-positive tumors were associated with positive regulation of biological processes such as RNA polymerase II promoter transcription and intercellular adhesion. These findings suggest that the epigenetic background can predict whether TLS-positive ccRCC tumors exhibit invasive behavior. Moreover, certain adhesion factors may recruit lymphocytes through a multi-step adhesion cascade mechanism, a critical step in TLS formation.

Beyond ccRCC, DNA methylation changes were also observed between TLS subgroups in EBV-negative gastric cancer patients (78). Specifically, the low-density TLS subgroup exhibited epigenetic silencing, primarily affecting immune-related transcription factors such as GFI1 and IRF4. As previously established, Tfh cells are localized at the center of TLS, marking the initial stage of TLS formation before GC appearance. Additionally, fibroblasts play a pivotal role in TLS development (79, 80).

Epigenetic regulation further influences TLS formation through SATB1, a nuclear matrix-binding protein that organizes chromatin loops and recruits epigenetic modifiers in immune cells, driving their phenotypes and inflammatory responses. Interestingly, Tfh cells express low levels of SATB1 (81). Supporting this, CHAURIO et al. demonstrated SATB1’s role as a “genomic organizer” in Tfh differentiation using a mouse model (82). Mechanistically, SATB1 induces the expression of ICOS, a key costimulatory factor for Tfh, by binding ~60 bp upstream of the ICOS transcription start site (83). Consequently, SATB1 deficiency leads to elevated ICOS levels, enhancing Tfh differentiation and increasing IL-21, CXCL13, and IgG1 antibody production by B cells. Simultaneously, SATB1 loss impairs naive T cell differentiation into Foxp3+ Treg cells, reducing their inhibitory effect on TLS formation. In summary, SATB1 silencing in CD4+ T cells suppresses the ICOS promoter, thereby promoting their differentiation into functional Tfh cells while inhibiting Treg generation. This creates a permissive microenvironment for B cell recruitment, chemokine activation, and TLS development.

## 5 TLS and tumor prognosis

TLS was first discovered in solid tumors of NSCLC patients, where the high density of these structures was associated with long-term survival (84). Subsequent studies revealed that the favorable prognostic value of TLS extends to a wide range of cancers, including CRC, lung cancer, breast cancer, melanoma, and pancreatic cancer, where TLS density consistently correlates with improved clinical outcomes (85–89). Notably, this prognostic significance is often independent of TNM staging.

Recently, ZHANG et al. identified TLS in cervical cancer for the first time, demonstrating that their abundance is influenced by tumor invasion depth, preoperative chemotherapy, HPV infection status, and PD-1 expression levels (90). Critically, TLS presence in these patients was linked to better prognosis. Beyond prognosis, TLS is increasingly recognized as a predictive biomarker for treatment response across multiple therapies, including chemotherapy, targeted therapy, and immunotherapy (91). For example, in TNBC, TLS density correlates with pathological complete response (pCR) after neoadjuvant chemotherapy and predicts superior outcomes for patients receiving targeted therapy. Similarly, in HER-2/neu+ tumors treated with trastuzumab, high TLS density is associated with improved DFS. In GIST patients, high TLS levels are linked to reduced imatinib resistance, lower recurrence rates, and prolonged OS (92). Furthermore, TLS shows promise in predicting response to immune checkpoint blockade (ICB). In melanoma, pre-treatment of CD20+ B cells within TLS coordinate tumor inflammation and enhance PD-1+ T cell activation upon anti-PD-1 therapy, thereby predicting ICB response and survival (93). This association is corroborated in NSCLC, soft tissue sarcoma, and urothelial carcinoma, where high TLS density correlates with better ICB outcomes (94–96). Collectively, these findings position TLS as a robust biomarker for patient stratification across cancer types and therapies.

The induction of TLS may represent an attractive therapeutic strategy for cancer treatment. Supporting this concept, in hepatoblastoma (HB) with APC mutations, TLS formation was significantly increased in paired tumor biopsies following cisplatin-based chemotherapy (97). This chemotherapy-induced TLS formation may promote immunogenic cell death (ICD), resulting in neoantigen release that is captured by DCs to initiate anti-tumor immune responses. Consistent with this immunostimulatory role, NSCLC patients treated with neoadjuvant anti-PD-1 therapy showed TLS enrichment that positively correlated with improved treatment response rates (98). However, the therapeutic impact appears context-dependent, as demonstrated by contrasting findings in squamous lung cancer where neoadjuvant chemotherapy impaired TLS maturation, caused GC loss, and abolished their prognostic value (99).

While numerous studies have demonstrated the association between high TLS density and improved long-term survival in various tumors, emerging evidence reveals a more complex relationship. In breast cancer, for instance, TLS presence correlated with prolonged survival in metastatic lesions (lung, liver, brain, and ovary), yet paradoxically, high TLS density was associated with worse outcomes in primary tumors (100). This dichotomy extends to other malignancies: Finkin et al. reported poorer prognosis in HCV-negative, non-alcoholic HCC patients with TLS, while Giraldo et al. identified a distinct TLS subtype (NTLS-DC) in clear cell renal cell carcinoma (ccRCC) that predicted shorter DFS and OS (101). Notably, these NTLS-DCs, located in tumor cores, exhibited low MHC II expression and contained immature DCs. Similarly, in advanced classical Hodgkin lymphoma, high TLS density unexpectedly increased mortality risk (102).

The dual role of TLS may stem from their functional heterogeneity. In aggressive cancers, TLS may foster immunosuppression through: (1) cytokine-mediated inhibitory microenvironments; (2) TLS-resident Tregs that dampen anti-tumor T cell responses; and (3) IL-10-secreting B cells that impair tumor-specific immunity. Moreover, tumor-specific antibodies generated in TLS might paradoxically suppress immunity via inhibitory Fc receptor signaling. However, whether these observations represent cancer-type-specific phenomena or broader patterns requires further investigation.

Spatial distribution adds another layer of complexity. In HCC, TLS within adjacent normal liver tissue correlated with higher recurrence risk or lacked prognostic value, in stark contrast to tumor-core TLS which predicted better outcomes (103). This spatial dichotomy was replicated in intrahepatic cholangiocarcinoma, where intratumoral TLS conferred survival benefits while peritumoral TLS associated with worse prognosis. Emerging evidence suggests this discrepancy may reflect compositional differences: intratumoral TLS harbor elevated Tfh and Treg populations compared to their peritumoral counterparts. To resolve these controversies, future studies should employ advanced animal models to elucidate TLS dynamics and microenvironmental interactions across tumor stages and locations.

## 6 TLS and immune therapy

The classical paradigm held that tumor-specific immune responses required DC migration from primary tumors to SLOs, where naïve CD4+ T cell activation occurred following tumor antigen presentation. However, emerging evidence challenges this view, demonstrating that efficient antigen presentation to T cells can occur directly within TLS in the TME, bypassing the need for DC trafficking to distant lymph nodes (104). This localized process enables more rapid and efficient antitumor immunity, with important implications for cancer immunotherapy. These findings have catalyzed significant interest in understanding TLS functions during immune checkpoint inhibitor (ICI) therapy. Unlike conventional cytotoxic therapies, ICB exerts antitumor effects by harnessing endogenous immunity a mechanism that highlights the critical role of TLS as specialized immune niches within the TIME (105). The TIME represents an intricate ecosystem of diverse immune cell populations that collectively influence tumor progression, treatment response, and clinical outcomes. As ectopic lymphoid organs, TLS positioned within or adjacent to tumors dramatically increase lymphocyte-tumor cell interactions, potentially amplifying local immune responses. This spatial advantage may explain their association with enhanced immunotherapeutic efficacy observed in multiple malignancies.

Mounting clinical evidence has established a bidirectional correlation between TLS presence and ICI efficacy: not only can TLS density predict ICI treatment outcomes, but conversely, ICI responsiveness may reflect TLS status. Compelling data demonstrate that ICI responders exhibit significantly higher intratumoral TLS densities compared to non-responders, suggesting that TLS-mediated T cell/B cell crosstalk potentiates antitumor immunity (106). Capitalizing on these prognostic associations, TLS induction has emerged as a promising immunotherapeutic strategy. Preclinical studies have validated the feasibility of generating ectopic TLS through localized delivery of lymphoid-organizing factors, including lymphotoxin, TNFα, LIGHT, CXCL13, CCL19, CCL21, and CXCL12 in murine models - providing a mechanistic foundation for clinical translation (107–111). Anatomically, TLS predominantly localize to stromal and peritumoral regions across multiple malignancies, including melanoma, NSCLC, HCC, and CSCC (112, 113). Notably, TLS occur with particularly high frequency in CSCC and CRCs, a phenomenon potentially attributable to the skin and gut’s specialized immune surveillance systems as barrier organs (114, 115). This anatomical predisposition suggests that TLS-rich microenvironments may amplify antitumor immunity through enhanced immune cell priming and activation.

While TLS can enhance tumor suppression, their presence may also trigger autoimmune reactions in normal tissues. As TLS form and mature within or around tumors, a large number of tumor-associated antigens are released. These antigens further activate immune cells, which can then migrate via the bloodstream and infiltrate normal tissues. Upon encountering self-antigens resembling tumor-associated antigens, these activated immune cells may mistakenly attack healthy tissues, leading to autoimmune pathology (116). Although data on such events are scarce, the potential for immune-related adverse outcomes cannot be ignored. Therefore, further research is needed to better harness TLS for antitumor immunity while minimizing autoimmune risks.

## 7 Standardization of TLS evaluation

Standardizing the qualitative and quantitative assessment of TLS is a crucial yet challenging task. Current evaluation methods primarily rely on histopathological approaches, utilizing immunohistochemistry and multiplex immunofluorescence techniques to identify the composition and spatial distribution of immune cell types within TLS. While this assessment approach is effective and intuitive to some extent, several major issues remain: First is subjectivity. Histopathological evaluation largely depends on the expertise of pathologists, leading to discrepancies in TLS interpretation among experts across different laboratories and research centers. Second, there is a lack of standardized assessment criteria for TLS. Currently, methods for evaluating TLS vary across research institutions, including inconsistencies in TLS quantification and the labeling of immune cells within TLS. These two major shortcomings limit the comparability and applicability of TLS research findings, hindering the clinical adoption of TLS as a predictive biomarker. Without unified standards, different evaluation results cannot be directly compared, making it difficult to translate research outcomes into practical clinical guidelines.

To address these issues, the following steps should be taken: First, it is essential to establish unified criteria for TLS evaluation, including standardization of immunohistochemical staining protocols, selection of lymphocyte markers and quantification methods for positivity, as well as clear criteria for assessing TLS maturity. Second, artificial intelligence (AI)-assisted analysis of digital pathology images should be employed to standardize the evaluation of TLS distribution density, maturity, and internal immune cell composition. This approach can reduce pathologist subjectivity, improve accuracy and reproducibility, and facilitate global adoption, thereby enhancing the translation of research findings into clinical practice. Finally, multicenter and multi-cohort validation is needed to refine TLS assessment standards. Testing and validating TLS evaluation methods across diverse populations and tumor types will improve the generalizability and reliability of the approach.

## 8 Conclusion

Recent studies highlighting the prognostic and predictive value of TLS in cancer have renewed interest in these ectopic immune aggregates as potential mediators of antitumor immunity. While direct evidence demonstrating the unique characteristics of immune responses originating within TLS remains scarce, cross-cancer analyses have consistently affirmed their clinical significance. However, the absence of standardized markers to define and characterize TLS represents a major translational hurdle that must be overcome to establish them as reliable biomarkers. To advance the field, we propose that a comprehensive definition of “TLS status” should integrate multidimensional characteristics, including cellular composition, spatial organization, maturation stage, and functional output. Such a framework would not only clarify their role in cancer immunity but also facilitate their clinical implementation as biomarkers. Importantly, systematic elucidation of TLS-associated molecular signatures through multi-omics approaches could further refine their prognostic and predictive value. Looking forward, a deeper mechanistic understanding of TLS functions—particularly their context-dependent roles in both antitumor immunity and autoimmune toxicity—will be essential to harness their full potential as therapeutic targets across cancer and chronic inflammatory diseases.
